# Brain Responses to Emotional Infant Faces in New Mothers and Nulliparous Women

**DOI:** 10.1038/s41598-020-66511-x

**Published:** 2020-06-12

**Authors:** Kaihua Zhang, Paola Rigo, Xueyun Su, Mengxing Wang, Zhong Chen, Gianluca Esposito, Diane L. Putnick, Marc H. Bornstein, Xiaoxia Du

**Affiliations:** 1 Department of Electronic Science, Fujian Provincial Key Laboratory of Plasma and Magnetic Resonance, Xiamen University, Xiamen, 361000 China; 2 Shanghai Key Laboratory of Magnetic Resonance and Department of Physics, School of Physics and Electronic Science, East China Normal University, Shanghai, 200062 China; 3 Department of Developmental Psychology and Socialisation, University of Padova, Padova, Italy; 4 Department of Special Education, Faculty of Education, East China Normal University, Shanghai, 200062 China; 5 Eunice Kennedy Shriver National Institute of Child Health and Human Development, NIH, Bethesda, MD USA; 6 Department of Psychology and Cognitive Science, University of Trento, Trento, Italy; 7 Psychology Programme, School of Social Sciences, Nanyang Technological University, Nanyang, Singapore

**Keywords:** Empathy, Social behaviour, Perception

## Abstract

The experience of motherhood is one of the most salient events in a woman’s life. Motherhood is associated with a series of neurophysiological, psychological, and behavioral changes that allow women to better adapt to their new role as mothers. Infants communicate their needs and physiological states mainly through salient emotional expressions, and maternal responses to infant signals are critical for infant survival and development. In this study, we investigated the whole brain functional response to emotional infant faces in 20 new mothers and 22 nulliparous women during functional magnetic resonance imaging scans. New mothers showed higher brain activation in regions involved in infant facial expression processing and empathic and mentalizing networks than nulliparous women. Furthermore, magnitudes of the activation of the left parahippocampal gyrus and the left fusiform gyrus, recruited during facial expression processing, were positively correlated with empathic concern (EC) scores in new mothers when viewing emotional (happy-sad) faces contrasted to neutral faces. Taken together, these results indicate that the experience of being a mother affects human brain responses in visual and social cognitive brain areas and in brain areas associated with theory-of-mind related and empathic processing.

## Introduction

When a woman experiences pregnancy and becomes a new mother, she undergoes a series of physical, psychological, and behavioral changes^1–4^. These changes help new mothers better adapt to their new role and enhance their responsiveness to children^5–8^ Reciprocally, infants communicate their needs and mental states mainly through vocalizations and facial expressions, which convey salient information that elicits affection and nurturing from adults^9–12^. A mother’s ability to accurately comment on her infant’s mental state is pivotal for the development of a secure attachment relationship and adaptive social functioning in children^13–18^. Thus, an appropriate understanding of the emotional content of infant stimuli by new mothers is of psychological and developmental significance.

Attuned mothers infer children’s needs, mental states, or motivations from the behaviors of their children and respond appropriately. Such social maternal abilities can be viewed as empathetic competence, which include appropriately perceiving infants’ feelings (called emotional or affective empathy) and recognizing infants’ needs (called cognitive empathy)^19^.

Empathy, the ability to share another’s feelings, is an important contributor to successful social interaction and allows for the prediction and understanding of another’s behavior and for reacting accordingly. The anterior cingulate cortex, with the medial prefrontal cortex and the anterior insula, and the temporo-parietal junction, with the somatosensory cortex, the superior temporal sulcus and the temporal pole, have been identified as key brain structures involved in empathy^20,21^. Recent research on empathy in humans has sought to dissociate its cognitive and emotional dimensions^22^. Specifically, cognitive empathy and perspective-taking tasks involve executive, working memory, and visual spatial processes (the dorsomedial and the dorsolateral prefrontal cortex, superior temporal gyrus, temporo-parietal junction and parietal lobule^23^). Emotional empathy involves brain regions related to affective and motor-motivational processes (such as the anterior cingulate cortex, thalamus, insula, fusiform gyrus, amygdala, somatosensory and motor cortices, and ventromedial prefrontal cortex^23^). Brain systems related to empathy overlap significantly and are recruited during the brain responses of parents to infant stimuli^24^.

Beyond empathy, mentalizing, the ability to understand the mental state of others that underlies behavior, crucially contributes to human social cognition and parenting as well^25^. Mentalizing network supports the parent’s capacity to read the infant’s nonverbal signals and infer the infant’s intentions^25,26^. A recent longitudinal study showed that the transition to motherhood induced in women changes in brain regions which partially overlap the theory-of-mind network^2^, which includes core areas of the temporo-parietal junction, medial prefrontal cortex, precuneus, anterior temporal lobes, and inferior frontal gyrus^27^. Other studies suggest that motherhood modulates human brain functions and enhances social cognition in response to infant cues^2,24,28,29^.

Moreover, parental status modulates female brain responses to emotional infant stimuli relevant to social adult-child social interactions. Functional magnetic resonance imaging (fMRI) has been used to examine mothers’ neural responses to emotional infant stimuli, such as infant sounds and facial expressions^19,30–33^. For example, mothers exhibit different brain activation patterns when responding to their own baby versus other babies^19,34,35^. Other studies have investigated brain responses to emotional infant stimuli in nulliparous women^34,36^, but few fMRI studies have focused on the impact of parental status in human brain responses to emotional infant stimuli through direct comparisons of new mothers and nulliparous women, as we do here.

Neuroimaging evidence using near-infrared spectroscopy suggests that distinguishing infant facial emotions increases activation in the right prefrontal cortex of mothers compared with non-mothers^37^. Rupp *et al*. reported reductions in both amygdala activation and subjective negative arousal in mothers (1–6 months postpartum) versus nulliparous women in response to negative infant images^38^. Parental status also impacts visually evoked potentials; the P110 early brain response over the left hemisphere is significantly larger in mothers versus childless women when examined during a judgment task of happy/distressed infant expressions; this finding may reflect a greater empathic response (or increased arousal) to infant facial expressions in mothers than in childless women^39^. Hayashi *et al*. found enhanced No-Go-P3 amplitudes in mothers compared with non-mothers in an emotional Go/No-go task, which indicated that mothers’ emotional regulatory processes may differ from those of non-mothers^40^. In general, infants’ emotional faces appear to be more salient for mothers than for non-mothers^41^.

The main purpose of the present study was to clarify differences between mothers and nulliparous women in response to emotional infant faces. In this study, we investigated whole brain functional responses of 20 primiparous mothers and 22 nulliparous women using happy, neutral, and sad emotional infant face stimuli during fMRI scans. We expected that emotional infant stimuli would induce different brain activation patterns in new mothers and nulliparous women. Specifically, we expected that the experience of being a mother would be reflected in different involvement of brain areas underlying (i) empathic processing (somatosensory, anterior cingulate cortex, and anterior insula)^20,42^ and (ii) theory of mind (medial prefrontal cortex, temporo-parietal junction, and precuneus)^27^, which play a pivotal role in mother-child social interaction. We also expected (iii) great involvement of cerebral regions previously reported in response to salient emotional infant faces in connection with both the processing of infant facial expressions and maternal memory (the insula, amygdala, hippocampus, parahippocampus, fusiform gyrus and occipital cortex).

## Materials and Methods

The experimental protocol was approved by the East China Normal University Committee on Human Research (No. HR201508001). Each participant firstly received an explanation about the experiment, then they are provided an informed consent form that was approved by the committee. All methods were conducted according to the principles outlined in the Declaration of Helsinki, including any relevant details.

### Participants

Twenty new mothers (M age 29.8 (SD = 2.0) years, range 27 to 33) and 22 nulliparous women (M age 26.5 (SD = 2.0) years, range 24 to 32) participated. The educational level was an average of 14.4 years (SD = 0.5) for new mothers and 15.6 years (SD = 0.6) for nulliparous women. Both the new mothers and the nulliparous women were right-handed, and all psychiatric and neurological diseases were excluded based on clinical examinations and MRI as well as structured interviews. All new mothers were first-time mothers, and their babies were aged 2 months to 11 months (M age 6.5 (SD = 3.0) months).

### fMRI Paradigm: affective picture task

Sixty infant face pictures were presented to evoke different emotions in the participants. The task included 20 happy infant faces, 20 neutral infant faces, and 20 sad infant faces selected from the Chinese affective picture system^43^. All pictures were previously assessed by 29 non-parental college student volunteers (M age = 25.1 ± 1.8 years old, 17 male and 12 female) from the same university using a 9-point Likert-type scale rating for valence and arousal. The three groups of pictures differed significantly in each valence dimension [*F* (2, 59) = 91.13, *p* < 0.0001; *M* ± SD: happy faces: 6.83 ± 0.25; neutral faces: 5.13 ± 0.20; sad faces: 2.76 ± 0.20] and arousal dimension [*F* (2, 59) = 16.8, *p* < 0.0001; *M* ± SD**:** happy faces: 6.34 ± 0.20; neutral faces: 4.28 ± 0.34; sad faces: 5.79 ± 0.21]. In each trial, the face picture was displayed for 2 s, and then a fixation crosshair was presented for 2 s, 4 s, or 6 s randomly. All trials were presented randomly. The fMRI task lasted 12 min 6 s. A SAMRTEC SA-9900 system (Shenzhen Sinorad Medical Electronics Inc., Shenzhen, China) was used to present all stimuli. This system could synchronize the stimuli presentation and the MRI scanner.

### fMRI Image acquisition

The MRI scanning was performed on a Siemens Trio Tim 3.0 Tesla MRI system at the Shanghai Key Laboratory of Magnetic Resonance (East China Normal University, Shanghai, China). We used a 12-channel head coil for the whole brain scanning. To minimize the head motion, the custom-fit foam pads were placed around the participants’ heads. High-resolution T_1_-weighted anatomical images were obtained using the 3-dimensional magnetization-prepared rapid-acquisition gradient-echo pulse sequence with the following acquisition parameters: voxel size = 1.0 × 1.0 × 1.0 mm^3^, field of view (FOV) = 256 × 256 mm^2^, slice thickness = 1 mm, number of slices = 192, echo time (TE) = 2.34 ms, repetition time (TR) = 2530 ms, inversion time (TI) = 1100, and flip angle = 7°. T_2_*-weighted blood oxygen level-dependent (BOLD) functional brain images were acquired using a gradient-echo echo-planar-imaging pulse sequence. The parameters are as follows: voxel size = 3.4 × 3.4 × 3.5 mm^3^, FOV = 220 × 220 mm^2^, slice thickness = 3.5 mm, 25% gap, number of slices = 33, TR = 2000 ms, TE = 30 ms, flip angle = 90°, and number of whole brain volumes = 363.

### fMRI Data analysis

Image processing of functional images was performed using Statistical Parametric Mapping (SPM12) software (http://www.fil.ion.ucl.ac.uk/spm/software/spm12). The 6 s dummy scans and first 4 s fixation were discarded to allow the signal to reach steady-state equilibrium. Data analysis included preprocessing procedures and statistical analyses. The preprocessing step contained slice timing correction, realignment, normalization to the Montreal Neurological Institute (MNI) space template, and spatial smooth. Slice timing correction was performed using the middle slice in time as a template. Spatial realignment was performed to correct for head motion. During realignment, 6 linear regressors were obtained describing the correction parameters applied at each volume. Data from participants who showed movements greater than 2 mm or 2 degrees were excluded from further analysis. To transform each participate brain into the MNI space, the functional images were co-registered with the structural images. Then functional images were spatially normalized to the MNI stereotaxic standard space using the parameters obtained from segmentation. The voxel size was interpolated to 3 mm × 3 mm × 3 mm. Finally, spatial smoothing with an 8 mm full-width half-maximum (FWHM) isotropic Gaussian kernel was performed on the functional images. The high-pass filter was 128 s. For each participate, the BOLD signal changes were convolved with a hemodynamic response function. Individual analysis (first level) was performed with a general linear model (GLM) including the 6 rigid body correction parameter regressors as covariates in the design matrix. In the first-level statistical analysis, an event statistical model was constructed, and three conditions were modeled for each subject (happy, neutral, and sad). The contrasts happy *minus* neutral, sad *minus* neutral, and happy *minus* sad were calculated for each participant. The resulting contrast images entered into the second-level group analysis (new mothers, nulliparous women). One-sample *t* tests were used to determine within-group activation. A flexible factorial design was used to study differences in brain activation, with group (new mothers vs. nulliparous women) as a between-subjects factor and condition (happy vs. neutral, sad vs. neutral, happy vs. sad) as a within-subjects factor. Participant (mother and child) age and education were added as covariates in all analysis. The BOLD value (extracted from contrast images of each group) of the clusters for which the ANOVA showed a significant effect is reported by post hoc analysis. The activation (spm(t) maps) were reported at *P* < 0.001 uncorrected at the voxel level and a cluster size threshold of *P* < 0.05 with false discovery rated (FDR) correction and were part of a 45-voxel cluster of contiguous significant voxels.

### Assessment of empathic abilities

We assessed participants’ empathic abilities using a multidimensional questionnaire, the Interpersonal Reactivity Index (IRI)^44,45^. The IRI is a multidimensional scale composed of 28 self-report items measuring cognitive and emotional dimensions of empathy^44,45^. The IRI assesses four dimensions; each dimension consists of seven items and the total scores for each subscale range from 0 to 28. The ‘fantasy’ (FS) scale measures the tendency of the participant to identify with fictitious characters in books and movies, and the ‘perspective-taking’ (PT) scale assesses the tendency to take the psychological point of view of others. The ‘empathic concern’ (EC) scale measures respondents’ prosocial feelings of warmth, compassion, and concern for others. The ‘personal distress’ (PD) scale measures self-oriented anxiety when observing others in distress. The PT and FS scales were designed to measure cognitive elements of empathy. The EC and PD scales were designed to measure emotional aspects of empathy. Higher subscale scores are associated with higher empathic tendencies ^44–47^. Participants completed the IRI independently after the MRI data acquisition.

### Exploratory correlation between fMRI data and IRI scores

The relationships between brain activity and IRI scores were analysis performed using SPSS 24.0 (SPSS, Chicago, Illinois). Specific activations identified in the interaction between groups and conditions were used to define regions of interests (ROIs). We extracted these individual mean beta values for Pearson correlation with IRI scores. SPM was used to determine these ROIs as masks. The ROIs signal extractor Marsbar toolbox (http://marsbar.sourceforge.net) was used for each ROI of every subject to extract mean parameter estimates for further correlation analysis in SPSS 24.0. The significant results were reported at p < 0.05 (two-tailed).

### fMRI results

First, we examine the main effect of parental status to obtain an overview of the brain regions that show significant variability in the two groups (mothers versus nulliparous women) with all infant emotional faces (SPM (F) maps; Table 1). Then, we detail the differences between mothers and nulliparous women in the following contrasts (SPM (t) maps): happy versus neutral faces, and sad versus neutral faces.

**Table 1:** Main effect of group.

No	Main effect of group	BA	Peak X	Peak Y	Peak Z	Z-value	F value	voxel number
1	**Right occipital lobe**	18/19/37	30	−63	−6	6.38	50.78	658
	right lingual gyrus							
	right middle occipital gyrus							
	right fusiform gyrus							
	right cuenus							
	right parahippocampa gyrus							
	right middle temporal gyrus							
	right inferior occipital gyrus right superior occipital gyrus							
2	**Left occipital lobe/cuneus**	18/19/37	−9	−96	15	5.93	43.08	782
	left middle occipital gyrus							
	left lingual gyrus							
	left fusiform gyrus							
	left inferior occipital gyrus							
	left parahippocampa gyrus							
3	**Left inferior frontal gyrus**	9	−39	6	33	5.15	31.53	86
	left middle frontal gyrus							
4	**Right middle frontal gyrus**	9	36	18	30	4.76	26.76	104
	right inferior frontal gyrus							

All the results reported reached cluster-level p < 0.05, with false discovery rate (FDR) correction for voxels surpassing a p < 0.001 initial voxel threshold.

### Effect of parental status (mothers versus nulliparous women)

**Figure 1:**
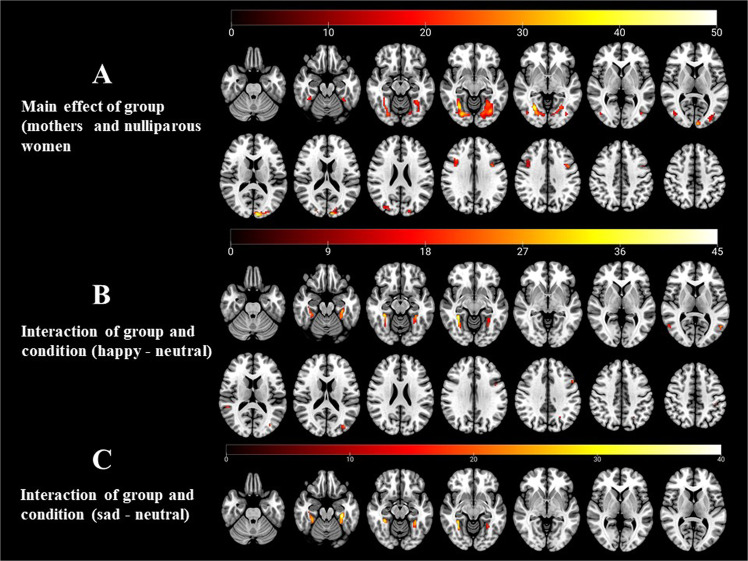
Brain activation map comparing new mothers to nulliparous women while viewing happy minus neutral infant faces and happy minus neutral infant faces. (**A**) Regions of activation included the bilateral occipital and frontal lobes. (**B**) Regions of activation included the bilateral parahippocampal gyri, bilateral superior and middle temporal gyri, left superior and inferior parietal lobule. (**C**) Regions of activation included the bilateral parahippocampal gyri. P < 0.05 with FDR corrected and cluster > 45.

Testing the main effect of group using a flexible factorial analysis, we found significant variability in the clusters centered in the right occipital lobe (extending to the lingual gyrus, superior, middle and inferior occipital gyri, cuneus, fusiform gyrus, middle temporal gyrus and parahippocampal gyrus); the right cuneus (extending to the lingual gyrus, middle and inferior occipital gyri, fusiform gyrus, and parahippocampal gyrus); the left inferior frontal gyrus (extending to the middle frontal gyrus); and the right middle frontal gyrus (extending to the inferior frontal gyrus) (Fig. 1A). For more details, see Table 1. The subsequent t-test revealed that mothers showed higher activation than non-mothers in all reported regions. No brain deactivation was found. (See Table S1 in the Supplementary Materials.)

### Happy-neutral faces contrast in mothers *versus* nulliparous women

When comparing happy faces with neutral faces (Fig. 1B, Table 2), two groups showed different activation in the right parahippocampal gyrus (extended to the fusiform gyrus), left parahippocampal gyrus (extended to the fusiform gyrus), left middle temporal gyrus (extended to middle occipital gyrus), left superior parietal lobule, left inferior frontal gyrus (extended to middle frontal gyrus), right superior temporal gyrus, right middle temporal gyrus (extended to superior temporal gyrus), left inferior parietal lobule (extended to the postcentral gyrus), and right cerebellum posterior lobe. In the supplementary materials, we report for the happy minus neutral contrast the specific cerebral activation in each group separately in Tables S2 and S3.

**Table 2:** Interaction of group and condition (happy – neutral).

No.	Group interaction of happy – neutral	Brodmann area	Peak X	Peak Y	Peak Z	Z-value	Cluster number
1	**Right parahippocampal gyrus**	37/36	33	−45	−9	6.03	204
	fusiform gyrus						
2	**Left parahippocampal gyrus**	37/36	−33	−33	−18	5.31	166
	fusiform gyrus						
3	**Left middle temporal gyrus**	39/19	−54	−63	6	5.16	69
	middle occipital gyrus						
4	**Left superior parietal lobule**	7	−18	−72	60	4.83	58
5	**Left middle temporal gyrus**	19	−36	−78	15	4.67	62
	Left middle occipital gyrus						
6	**Left inferior frontal gyrus** Left middle frontal gyrus	9	−54	12	36	4.66	58
7	**Right superior temporal gyrus**	—	51	−39	12	4.46	47
8	**Right middle temporal gyrus** Right superior temporal gyrus	39	54	−66	6	4.35	49
9	**Left inferior parietal lobule** Left postcentral gyrus	40	−39	−33	42	3.92	47
10	Right cerebellum posterior lobe	—	24	−66	−30	3.83	45

All the results reported reached cluster-level p < 0.05, with false discovery rate (FDR) correction for voxels surpassing a p < 0.001 initial voxel threshold.

### Sad-neutral faces contrast in mothers *versus* nulliparous women

In the contrast of sad and neutral faces (Fig. 1C, Table 3), two groups showed different activation in the left parahippocampal gyrus (extended to the left fusiform gyrus, and left cerebellum anterior and posterior lobes), and right parahippocampal gyrus (extended to the right fusiform gyrus and left cerebellum anterior lobe). In the supplementary materials, we report for the sad minus neutral contrast the specific cerebral activation in each group separately in Tables S2 and S3.

**Table 3:** Interaction of group and condition (sad – neutral).

No.	Group interaction of sad – neutral	Brodmann area	Peak X	Peak Y	Peak Z	Z-value	Cluster number
1	**Left parahippocampal gyrus**	37/36	−30	−30	−21	5.71	165
	left fusiform gyrus						
	left cerebellum anterior and posterior lobes						
2	**Right parahippocampal gyrus**	37/36/20	33	−45	−9	5.46	197
	right fusiform gyrus left cerebellum anterior lobe						

All the results reported reached cluster-level p < 0.05, with false discovery rate (FDR) correction for voxels surpassing a p < 0.001 initial voxel threshold.

### IRI scores and results from group comparisons

The four dimensions (FS, PT, EC, PD) and the total IRI scores were as follows: PT 12.2 ± 2.9, EC 18.4 ± 4.3, FS 13.2 ± 3.7 (mean ± SD), PD 10.0 ± 3.4, and total 53.8 ± 9.3 in new mothers; PT 11.8 ± 3.0, EC 15.5 ± 2.3, FS 13.6 ± 4.2, PD 9.4 ± 2.8 and total 50.3 ± 6.4 for nulliparous women. T-test analysis revealed that new mothers showed significantly higher EC scores than nulliparous women (T = 2.78, P = 0.008), and there was no significant difference between the two groups in the PT, FS, PD, and total IRI scores (see Table 4). Statistical calculations were conducted using SPSS 24.0 (SPSS, Chicago, Illinois) for Windows.

**Table 4:** The Interpersonal Reactivity Index (IRI) of new mothers and nulliparous women.

Dimensions	Mean ± SD		T value Sig. (2-tailed)	
	New mothers	Nulliparous women		
PT	12.2 ± 2.9	11.8 ± 3.0	0.42	0.68
EC	18.4 ± 4.3	15.5 ± 2.3	2.78	**0.008**
FS	13.2 ± 3.7	13.6 ± 4.2	−0.36	0.72
PD	10.0 ± 3.4	9.4 ± 2.8	0.61	0.54
Total	53.8 ± 9.3	50.3 ± 6.4	1.43	0.16

PT: perspective taking; FS: fantasy score; EC: empathic concern scale; PD: personal distress. Comparisons among groups were made by an independent samples T test.

### Correlations between fMRI data and IRI scores

**Figure 2:**
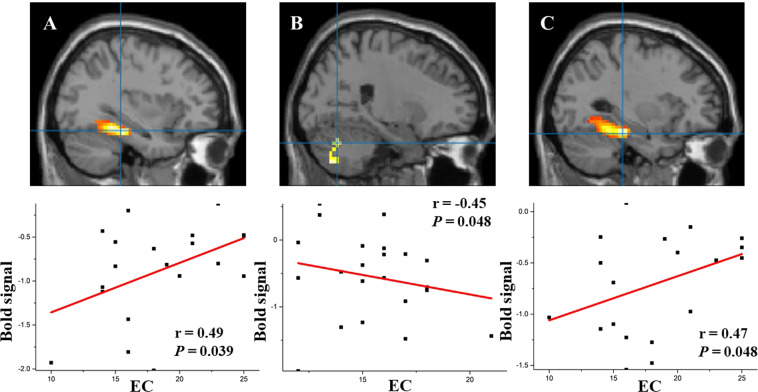
Correlations between BOLD fMRI activity and IRI scores in mothers and nulliparous women. (**A**) correlation between BOLD activity and EC score in new mothers for happy faces minus neutral faces. (**B**) correlation between BOLD fMRI activity and EC score in nulliparous women for happy faces minus neutral faces. (**C)**: correlation between BOLD activity and EC score in new mothers during sad faces minus neutral faces.

When comparing happy faces with neutral faces, the activation of the left parahippocampal gyrus extending to the left fusiform gyrus (−33, −33, −18; r = 0.49, *P* = 0.039, uncorrected) were positively correlated with the EC score in new mothers (see Fig. 2A), and the activation of the right cerebellum posterior lobe (24, −66, −30; r = −0.45, *P* = 0.048, uncorrected) were negatively correlated with the EC score in nulliparous women (see Fig. 2B).

When comparing sad faces with neutral faces, the activation of the left parahippocampal gyrus extending to the left fusiform gyrus and left cerebellum anterior/posterior lobes (−30, −30, −21; r = 0.47, P = 0.048, uncorrected) were positively correlated with the EC score in the new mothers group (see Fig. 2C), and no activations were found in nulliparous women that were related to the EC score.

## Discussion

Babies and young infants communicate their needs and physiological states mainly through salient emotional cues. Research shows that new mothers undergo a series of adaptive changes that support them in better adapting to their new role^2,10,48^. The extant literature suggests that some aspects of cognition are enhanced during pregnancy and the early postpartum period. The main purpose of the present study was to investigate differences in brain responses to emotional infant faces in new mothers and nulliparous women. We hypothesized that emotional infant stimuli induce different brain activation patterns in new mothers and nulliparous women in brain areas related to infant facial expression processing and empathic and mentalizing networks. Main effects of parental status are consistent with our hypotheses. Considering all the emotional infant pictures together, our results indicate that new mothers and nulliparous women show different responses in the social cognitive brain areas related to empathic processing and theory of mind, namely the bilateral inferior and middle frontal gyri, right middle temporal gyrus, bilateral lingual gyri, bilateral fusiform gyri, bilateral cuneus, bilateral parahippocampal gyri, and bilateral middle and inferior occipital gyri. Additionally, in new mothers activation of the left parahippocampal gyrus and left fusiform gyrus, and left cerebellum anterior/posterior lobes was positively correlated with the EC score when viewing emotional faces minus neutral faces, while in nulliparous women activation of the right cerebellum posterior lobe was negatively correlated with the EC score when viewing emotional faces minus neutral faces (see Fig. 2). Taken together, the results clearly suggest that new mothers and nulliparous women show different brain responses for emotional infant cues.

Specifically, according to the first expectation, in response to emotional infant faces, mothers showed greater activation in the somatosensory cortex, the medial prefrontal cortex, the middle and inferior temporal gyri, which are proposed to be part of the brain structures involved in emotional empathy (see Table 2 and Table S1)^20,21,23^. The greater response of these brain regions to emotional infant faces in new mothers may reflect a tagging of the infant stimuli as the current focus, which promotes further processing in maternal brain networks. Compared to nulliparous women, emotional infant faces can elicit higher empathic responsiveness in new mothers, which likely plays a critical role in facilitating caregiving behavior toward the infant^49^.

In partial accord to the second expectation, mothers show greater brain activation in the medial prefrontal cortex, inferior frontal gyrus extended to the precentral gyrus, the middle and inferior temporal cortices, which are involved in the theory-of-mind networks and its extended brain regions (see Table 2 and Table S1)^27,50^. Additionally, the inferior frontal gyrus/premotor cortex are known as mirror neurons and play a role in understanding the intentions of others and, for our purpose, in sharing the emotions of others^51^. Despite, in the present study, no significant temporo-parietal junction activation were found, we found elevated activations in key regions of the theory-of-mind and social cognition in new mothers, compared to the nulliparous women, in response to either happy or sad infant faces (Table S2).

Finally, consistent with the third expectation, the brain areas involved in visually processing salient emotional stimuli, the occipital lobe, lingual gyrus, middle and inferior occipital gyri, cuneus, fusiform gyrus, and parahippocampal gyrus, showed greater involvement. Furthermore, a correlation between the fMRI data and IRI scores of new mothers showed that activation of the left parahippocampal gyrus and left fusiform gyrus was positively correlated with the EC score when viewing emotional faces minus neutral faces. The lingual gyrus, fusiform gyrus, and parahippocampal gyrus have been reported to be associated with the processing of emotional faces (see Table 2 and Table S1)^53,54^. The fusiform gyrus is a key structure for functionally specialized computations of high-level vision, such as face perception, object recognition, and reading^55^, and it is involved in processing emotional expressions^56,57^. The parahippocampal cortex has been associated with many cognitive processes, including visuospatial processing, emotion processing and episodic memory^58^. The fusiform gyrus is near the parahippocampal gyrus, and these regions have been shown to be more active during the perception of emotional faces than the perception of neutral faces^53,54^. In the present study, mothers showed weaker deactivation than nulliparous women in these two areas when viewing happy/sad faces minus neutral faces, as found in the present analysis. This finding indicates that there is less inhibition in visual processing among new mothers when viewing emotional faces, which suggests that new mothers may be more sensitive than nulliparous women to infant emotional stimuli^59^.

In the individual contrasts that examined the specific brain response to positive and negative infant faces separately, mothers and non-mothers showed differentiations consistent with our expectations. When comparing happy or sad faces with neutral faces, mothers showed different activation in brain regions underlie the empathy processing (the postcentral gyrus)^20,42^, theory-of-mind (middle and inferior frontal gyri, superior and middle temporal gyri, inferior parietal lobule, and angular gyrus)^27^, as well as infant facial expressions processing and maternal memory (parahippocampal gyrus, fusiform gyrus, and middle occipital gyrus)^58^.

In line with the literature, new mothers and nulliparous women also showed similar brain activation in the visual areas, limbic, temporo-parietal, prefrontal, and subcortical areas as well as the cerebellum, as reported in previous studies^53,57,60^. However, an intriguing result concerns the fact that new mothers and nulliparous women also differed in the extension (areas) of brain activation; activated brain regions were more focal in new mothers, while nulliparous women showed more diffuse activation. There is evidence that smaller volume recruitment of task-related regions can reflect an increased ‘neural efficiency’ in the brains of individuals with higher competences in the task^61,62^. In fact, in our study, primiparous mothers showed increased empathic concern (EC score), which involves prosocial feelings of warmth, compassion, and concern for others (see Table 1). More focal brain activity in new mothers may reflect maternal experience in childcare, and therefore great expertise and inclination in processing emotional cues from children. In accord with the last point, the present study showed that the brain response to infant facial expression processing (such as the left parahippocampal gyrus, the left fusiform gyrus, the left middle occipital gyrus, and the left cerebellum anterior/posterior lobes) correlates with the empathic concern for others in mothers but not in nulliparous women. Indeed, we found that greater empathic concern of mothers is associated with a lower magnitude of activation in brain structures underlying the mentalizing network in response to positive and negative emotional faces contrasted to neutral faces, which can potentially reflect social expertise of motherhood.

Empathy and the mentalizing networks are considered core components of the human parental brain^24,52^, and maternal sensitivity has been shown to be important for secure parent–infant attachment and for the development of the child’s own social cognitive functions^13,63^. Research suggests that mother’s ability to comment accurately on an infant’s mental states predicts security of attachment at 12 months^13^. In fact, social cognitive abilities are important for providing adequate maternal care and successfully rearing offspring in the daily social environment. High social cognitive ability can help new mothers understand their children’s needs, decode social stimuli that may lead to potential threats, and promote the integration of mothers and babies. In our study, primiparous mothers showed increased empathic concern (EC score) and weaker activation and deactivation than nulliparous women when viewing infants’ emotion faces, which may suggest that new mothers may sense infant emotions, such as happiness or sadness, more easily than nulliparous women. The enhanced ability to encode emotional faces may be an evolutionary adaptation to prepare women for the protective and nurturing demands of motherhood by increasing their general emotional sensitivity^28^.

While our research revealed that new mothers are more sensitive to infant emotional stimuli, our study had several limitations. We focused only on task functional changes and did not examine structural abnormalities. In a future study, we will assess structural changes using voxel-based morphometry or diffusion tensor imaging analyses. Furthermore, the nulliparous women group and the new mother group were not age-matched. In general, the new mothers were older than the nulliparous women, and older nulliparous women are very rare in China. Thus, in the data analysis, we used each subject’s age as a covariate. Besides, each condition of the stimulation task contains 20 trials, and the limited number of trials may have affected the statistical power. For example, there is no significant difference between the two groups when comparing happy vs sad faces conditions at the present statistical threshold.

## Conclusion

Taken together, our results indicated that motherhood (new mothers versus nulliparous women) affects human brain responses to emotional infant stimuli in brain areas involved in infant’s face visual processing and in social cognitive brain areas that are related with empathic processing and theory-of-mind. The results of this study indicate that being a mother is associated with alterations in social cognitive function, which may serve an adaptive purpose for pending motherhood.

## Supplementary information

Supplementary Materials.
