# Supplementary material for: Brain Responses to Emotional Infant Faces in New Mothers and Nulliparous Women
Source: Sci Rep. 2020 Jun 12;10:9560. doi: 10.1038/s41598-020-66511-x (PMC7293211; doi:10.1038/s41598-020-66511-x)
Supplement: Supplementary file 1 — Supplementary Materials. [file 41598_2020_66511_MOESM1_ESM.pdf]

Brain Responses to Emotional Infant Faces in New Mothers and Nulliparous Women

Kaihua Zhang[1, 2], Paola Rigo[3], Xueyun Su[4], MengxingWang[2], Zhong Chen[1], Gianluca Esposito[6,7], Diane L. Putnick[5], Marc H. Bornstein[5,] Xiaoxia Du[2]*

[1]Department of Electronic Science, Xiamen University, Xiamen 361000, China;
[2]Shanghai Key Laboratory of Magnetic Resonance and Department of Physics, School of Physics and Electronic Science, East China Normal University, Shanghai 200062, China;
[3]Department of Developmental Psychology and Socialisation, University of Padova, Padova, Italy
[4]Department of Special Education, Faculty of Education, East China Normal University, Shanghai 200062, China;
[5]Eunice Kennedy Shriver National Institute of Child Health and Human Development, NIH, Bethesda, MD, USA;
[6]Department of Psychology and Cognitive Science, University of Trento, Trento, Italy;
7 Psychology Programme, School of Social Sciences, Nanyang Technological University, Singapore

Correspondence and requests for materials should be addressed to Xiaoxia Du (xxdu@phy.ecnu.edu.cn).

STable 1. Main effect of group with T contrast (mother > non-mother).

No	Main effect of group	Peak X	Peak Y	Peak Z	Z-value	T-value	voxel number
1	**Right occipital lobes**	30	-63	-6	6.49	7.13	792
	right middle occipital gyri						
	right lingual gyri						
	right cuenus						
	right fusiform gyri						
	right parahippocampa gyri						
	right middle temporal gyri						
	right inferior occipital gyri						
	right superior occipital gyrus						
	right inferior temporal gyrus						
2	**Left middle occipital gyrus**	-9	-96	15	6.05	6.56	915
	left middle occipital gyrus						
	left cuneus						
	left lingual gyrus						
	left fusiform gyrus						
	left parahippocampal gyrus						
	left inferior occipital gyrus						
	left cerebellum anterior lobe						
3	**Left inferior frontal gyrus**	-39	6	33	5.28	5.62	102
	Left middle frontal gyrus						
	Left precentral gyrus						
4	**Right middle frontal gyrus**	36	18	30	4.90	5.17	130
	Right inferior frontal gyrus						

X, Y, Z = MNI coordinates; $p < 0.001$ (voxel level), and FDR were corrected to $p < 0.05$ at the cluster level.

STable 2. Significant activation when new mothers view happy/Sad to neutral faces.

Cluster	Brain regions	Cluster size	X	Y	Z	Z value
**Increased activation: happy faces > neutral faces**						
1	**Right inferior occipital gyrus**	279	42	-78	-9	5.77
	right middle occipital gyrus					
	right middle temporal gyrus					
	right inferior occipital gyrus					
	right inferior temporal gyrus					
**Deactivation: neutral faces > happy faces**						
1	**Left cerebellum posterior lobe**	658	-27	-54	-18	6.08
	left lingual gyrus/BA18					
	left fusiform gyrus/BA19					
	left parahippocampal gyrus					
	left cerebellum anterior lobe					
2	**Left middle occipital gyrus**	907	-33	-87	12	5.99
	left precuneus					
	left inferior parietal lobule					
	left cuneus					
	left postcentral gyrus					
	left superior parietal lobule					
	left middle temporal gyrus					
	left superior occipital gyrus					
3	**Right cerebellum anterior lobe**	409	27	-48	-18	5.77
	right lingual gyrus/BA18					
	right fusiform gyrus					
	right parahippocampal gyrus					
	right cerebellum posterior lobe					
	right cuneus					
4.	**Right middle occipital gyrus**	198	33	-84	12	5.5
	right middle temporal gyrus					
	right cuneus					
	Right superior occipital gyrus					
5	**Right precuneus**	132	21	-57	42	3.91

	right superior parietal lobule					
**Increased activation: sad faces > neutral faces**						
1	**Right middle occipital gyrus/BA37**	275	48	-69	0	5.15
	right inferior temporal gyrus					
	right middle temporal gyrus					
	right inferior occipital gyrus					
	right superior temporal gyrus					
**Deactivation: neutral faces > sad faces**						
1	**Left middle occipital gyrus**	700	-30	-87	12	6.14
	left precuneus					
	left superior parietal lobule					
	left cuneus					
	left middle temporal gyrus					
	left superior occipital gyrus					
2	**Left cerebellum posterior lobe**	453	-27	-54	-15	5.80
	left fusiform gyrus/BA19					
	left lingual gyrus/BA18					
	left cerebellum anterior lobe					
	left parahippocampal gyrus					
	left middle occipital gyrus					
	left middle temporal gyrus					
3	**Right middle occipital gyrus**	399	33	-84	12	5.66
	right precuneus					
	right superior parietal lobule					
	right middle temporal gyrus					
	right cuneus					
	right superior occipital gyrus					
4	**Right cerebellum posterior lobe**	321	27	-57	-15	5.14
	right fusiform gyrus					
	right cerebellum anterior lobe					
	right parahippocampal gyrus					
	right lingual gyrus/BA18					
	right cuneus					

X, Y, Z = MNI coordinates; $p < 0.001$ (voxel level), and FDR were corrected to $p < 0.05$ at the cluster level.

STable 3. Significant activation when nulliparous women view happy/sad to neutral faces.

Cluster	Brain regions	Cluster size	X	Y	Z	Z value
**Increased activation: happy faces > neutral faces**						
1	**Right middle occipital gyrus**	379	51	-69	6	5.35
	right superior temporal gyrus					
	right middle temporal gyrus					
	right inferior temporal gyrus					
	right inferior occipital gyrus					
2	**Right cuneus**	368	6	-69	30	4.90
	right precuneus					
	right cingulate gyrus					
	right posterior cingulate gyrus					
3	**Left middle occipital gyrus**	178	-48	-75	3	4.86
	left middle temporal gyrus					
	left superior temporal gyrus					
	left inferior occipital gyrus					
**Deactivation: neutral faces > happy faces**						
1	**Right cerebellum posterior lobe**	581	27	-54	-18	6.74
	right lingual gyrus/BA18					
	right parahippocampal gyrus					
	right fusiform gyrus/BA19					
	right cerebellum anterior lobe					
	right cuneus					
2	**Left parahippocampal gyrus**	634	-33	-30	-24	6.26
	left lingual gyrus					
	left fusiform gyrus					
	left cerebellum anterior lobe					
	left cerebellum posterior lobe					
3	**Right middle occipital gyrus**	927	-33	-90	15	5.73
	right precuneus					
	right inferior parietal lobule					
	left superior parietal lobule					
	left postcentral gyrus					
	left cuneus					

	left middle temporal gyrus					
	left superior occipital gyrus					
4	**Right middle occipital gyrus**	527	33	-78	9	5.19
	right precuneus					
	right middle temporal gyrus					
	right superior parietal lobule					
	right superior occipital gyrus					
	right cuneus					
5	**Left middle occipital gyrus**	**76**	-48	-60	-9	5.10
	left middle temporal gyrus					
	left inferior temporal gyrus					
6	**Right postcentral gyrus**	69	45	-27	45	4.73
	right inferior parietal lobule					
7	**Left middle frontal gyrus**	72	-54	12	36	4.15
	left inferior frontal gyrus					
**Increased activation: sad faces > neutral faces**						
1	**Left middle occipital gyrus/BA37**	226	-51	-75	3	5.90
	left superior temporal gyrus					
	left middle temporal gyrus					
	left inferior occipital gyrus					
2	**Bilateral middle occipital gyrus/BA37**	1114	51	-72	0	5.01
	bilateral precuneus					
	bilateral superior temporal gyrus					
	bilateral middle temporal gyrus					
	bilateral posterior cingulate gyrus					
	bilateral cuneus					
	bilateral lingual gyrus					
	bilateral inferior occipital gyrus					
	bilateral cerebellum anterior Lobe					
	bilateral parahippocampa gyrus					
	bilateral corpus callosum					
	bilateral inferior temporal gyrus					
**deactivation: neutral faces > sad faces**						
1	**Right fusiform gyrus**	638	33	-45	-12	6.24
	right lingual gyrus					

	right cerebellum posterior lobe					
	right parahippocampa gyrus					
	right cerebellum anterior lobe					
	right cuneus					
2	**Left precuneus**	1090	-24	-63	36	6.17
	left superior parietal lobule					
	left inferior parietal lobule					
	left middle occipital gyrus					
	left cuneus					
	left postcentral gyrus					
	left middle temporal gyrus					
	left superior occipital gyrus					
3	**Left fusiform gyrus**	554	-27	-66	-12	5.99
	left lingual gyrus					
	left parahippocampa gyrus					
	left cerebellum anterior lobe					
	left cerebellum posterior lobe					
4	**Right middle occipital gyrus**	598	33	-78	12	5.64
	right precuneus					
	right superior parietal lobule					
	right middle temporal gyrus					
	right cuneus					
	right superior occipital gyrus					
	right inferior parietal lobule					
	right angular gyrus					
5	**Left middle occipital gyrus**	92	-48	-63	-9	5.53
	left middle temporal gyrus					
	left inferior temporal gyrus					
6	**Right middle frontal gyrus**	61	24	-6	48	5.33
7	**Left middle frontal gyrus**	95	-24	3	54	4.26
8	**Left middle frontal gyrus**	64	-42	27	18	4.14
9	**Left inferior frontal gyrus**	74	-51	9	36	4.13
	left middle frontal gyrus					
	left precentral gyrus					

X, Y, Z = MNI coordinates; $p < 0.001$ (voxel level), and FDR were corrected to $p < 0.05$ at the cluster

level.
